# Coupling of polymerase and carrier lipid phosphatase prevents product inhibition in peptidoglycan synthesis

**DOI:** 10.1016/j.tcsw.2018.04.002

**Published:** 2018-04-19

**Authors:** Víctor M. Hernández-Rocamora, Christian F. Otten, Atanas Radkov, Jean-Pierre Simorre, Eefjan Breukink, Michael VanNieuwenhze, Waldemar Vollmer

**Affiliations:** a Centre for Bacterial Cell Biology, Institute for Cell and Molecular Biosciences, Newcastle University, Richardson Road, Newcastle upon Tyne NE2 4AX, UK; b Department of Chemistry, Indiana University, 800 E. Kirkwood Avenue, Bloomington, IN 47405-7102, USA; c Institut de Biologie Structurale, Université Grenoble Alpes, Grenoble, France; d Membrane Biochemistry and Biophysics, Bijvoet Centre for Biomolecular Research, University of Utrecht, Padualaan 8, 3584 Utrecht, The Netherlands

**Keywords:** Peptidoglycan, Penicillin-binding protein, Undecaprenyl phosphate, BacA, PgpB, PBP1B

## Abstract

Peptidoglycan (PG) is an essential component of the bacterial cell wall that maintains the shape and integrity of the cell. The PG precursor lipid II is assembled at the inner leaflet of the cytoplasmic membrane, translocated to the periplasmic side, and polymerized to glycan chains by membrane anchored PG synthases, such as the class A Penicillin-binding proteins (PBPs). Polymerization of PG releases the diphosphate form of the carrier lipid, undecaprenyl pyrophosphate (C55-*PP*), which is converted to the monophosphate form by membrane-embedded pyrophosphatases, generating C55-*P* for a new round of PG precursor synthesis. Here we report that deletion of the C55-*PP* pyrophosphatase gene *pgpB* in *E. coli* increases the susceptibility to cefsulodin, a β-lactam specific for PBP1A, indicating that the cellular function of PBP1B is impaired in the absence of PgpB. Purified PBP1B interacted with PgpB and another C55-*PP* pyrophosphatase, BacA and both, PgpB and BacA stimulated the glycosyltransferase activity of PBP1B. C55-*PP* was found to be a potent inhibitor of PBP1B. Our data suggest that the stimulation of PBP1B by PgpB is due to the faster removal and processing of C55-*PP*, and that PBP1B interacts with C55-*PP* phosphatases during PG synthesis to couple PG polymerization with the recycling of the carrier lipid and prevent product inhibition by C55-*PP*.

## Introduction

**Fig. 1:**
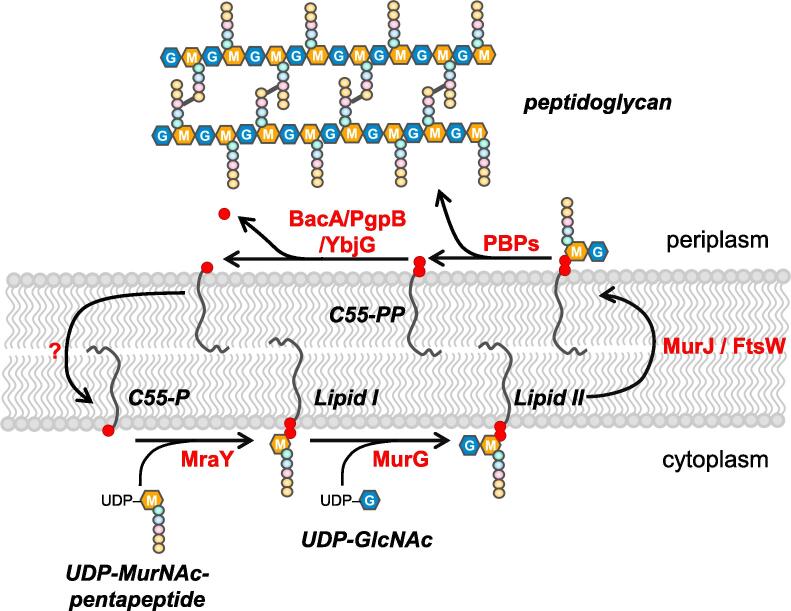
Membrane-associated steps in PG synthesis in *Escherichia coli*. The precursor lipid II is synthesized at the inner leaflet of the cytoplasmic membrane in two steps. MraY transfers Mur*N*Ac(pentapeptide)-phosphate from UDP-Mur*N*Ac-pentapeptide to the carrier lipid undecaprenyl phosphate (C55-*P*) to form lipid I. MurG transfers Glc*N*Ac from UDP-Glc*N*Ac to lipid I producing lipid II. Lipid II is then flipped to the outer leaflet of the cytoplasmic membrane and polymerized by glycosyltransferase activities of PBPs, releasing undecaprenyl pyrophosphate (C55-*PP*). C55-*PP* is dephosphorylated by the periplasmic phosphatases BacA, PgpB and YbjG, and transported back to the inner leaflet to start a new cycle.

Peptidoglycan (PG) is an essential component of the bacterial cell envelope protecting the cells from bursting due to its turgor and maintaining the shape of the cell. PG forms a continuous structure, called sacculus, consisting of glycan chains of alternating *N*-acetylglucosamine (Glc*N*Ac) and *N*-acetylmuramic acid (Mur*N*Ac) residues connected by short peptides (Vollmer et al., 2008). Growing and dividing cells enlarge their sacculus by incorporation of nascent PG that is synthesized from the precursor lipid II (Fig. 1). The two final PG precursors contain a carrier lipid, undecaprenyl phosphate (C55-*P*) and are assembled at the inner leaflet of the cytoplasmic membrane. First, the transferase MraY produces lipid I from the soluble nucleoside-precursors UDP-Mur*N*Ac-pentapeptide and the carrier lipid, C55-*P* (Barreteau et al., 2008). Second, MurG catalyses the transfer of a Glc*N*Ac moiety from UDP-Glc*N*Ac to lipid I, forming lipid II (Liu & Breukink, 2016). Lipid II is then translocated across the cytoplasmic membrane by proteins belonging to the SEDS (shape, elongation, division and sporulation) family, MurJ or both, and polymerized by PG glycosyltransferases (GTases) (Ruiz, 2015, Leclercq et al., 2017, Mohammadi et al., 2011).

*Escherichia coli* has six PG synthases of which two semi-redundant bifunctional GTase-TPases, the Penicillin-binding proteins (PBPs) PBP1A and PBP1B (Yousif et al., 1985), play major roles in PG synthesis during cell elongation and division. These enzymes are anchored to the cytoplasmic membrane by a single *N*-terminal transmembrane helix and are engaged in a number of protein-protein interactions within dynamic multi-protein complexes presumably to regulate their activities (Typas et al., 2012). PBP1A and PBP1B are both regulated by a cognate outer membrane-anchored lipoprotein – LpoA and LpoB, respectively (Typas et al., 2010, Paradis-Bleau et al., 2010) – which is needed to activate the PBP through the PG layer presumably in response to the properties of the pores in the PG layer (Typas et al., 2012). The stimulation of PBP1B by LpoB is modulated by CpoB and TolA to coordinate outer membrane constriction with the synthesis of septal PG (Gray et al., 2015).

The polymerization of new glycan strands occurs at the outer leaflet of the cytoplasmic membrane and releases the carrier lipid in the diphosphate form, undecaprenyl pyrophosphate (C55-*PP*). C55-*PP* is also synthesised *de novo* at the inner leaflet of the cytoplasmic membrane by UppS (Teng & Liang, 2012), and needs to be dephosphorylated to C55-*P* which is the universal carrier lipid not only for PG precursors, but also for precursors for the O-antigen moiety of lipopolysaccharide (LPS), the enterobacterial common antigen and colanic acid (Manat et al., 2014).

In *E. coli*, C55-*PP* molecules can be dephosphorylated on the periplasmic side of the membrane by four undecaprenyl pyrophosphate phosphatases (UPPs), BacA (UppP) and three members of the type 2 phosphatidic acid phosphatase family (PAP2), PgpB, YbjG and LpxT (Fig. 1). Their collective activity is essential. However, only PgpB, YbjG and BacA can sustain growth in the absence of all other phosphatases (El Ghachi et al., 2005). LpxT has also phosphotransferase activity, transferring a phosphate from C55-*PP* to the LPS precursor lipid A (Touzé et al., 2008b). BacA is widely conserved in bacteria (Bickford & Nick, 2013) and contributes to ∼75% of the UPP activity in *E. coli* (El Ghachi et al., 2005). Whilst BacA is specific for C55-*PP*, PgpB has a more relaxed substrate specificity, also participating in the phosphatidylglycerol metabolism (Touzé et al., 2008a, Fan et al., 2014).

Here we show that the deletion of *pgpB* increases the susceptibility of *E. coli* cells to cefsulodin, indicating an impaired function of PBP1B. PgpB and PBP1B formed a complex *in vitro* as demonstrated by pull down and surface plasmon resonance experiments. C55-*PP* inhibited the GTase activity of PBP1B and both, BacA and PgpB stimulated PBP1B in detergents and when reconstituted in membranes. Hence, our data suggest that the GTase and C55-*PP* dephosphorylation reactions are coupled and that this coupling prevents the product inhibition of the GTase active site.

## Materials and methods

### Chemicals

[^14^C]Glc*N*Ac-labelled lipid II and dansylated lipid II were prepared as published (Breukink et al., 2003, Bertsche et al., 2005). Unlabelled lipid II was purchased from the BACWAN facility (Warwick, UK). Polar lipid extract from *E. coli*, 1,2-dipalmitoleoyl-*sn*-glycero-3-phosphocholine (DOPC), 1-palmitoyl-2-oleoyl-*sn*-glycero-3-phospho-(1′-*rac*-glycerol) (POPG) and tetraoleoyl cardiolipin (TOCL) were obtained from Avanti Polar Lipids (Alabaster, USA). Lipids were resuspended in chloroform:methanol (2:1) at a concentration of 20 g/L, aliquoted and stored at −20 °C. Farnesyl pyrophosphate (C15-*PP*), isopentenyl pyrophosphate (C5-*PP*), Triton X-100, phenylmethylsulfonyl fluoride (PMSF), protease inhibitor cocktail (PIC) and β-mercaptoethanol were from Millipore Sigma. Undecaprenyl monophosphate diammonium salt (C55-*P*) was obtained from Larodan (Solna, Sweden). Upon arrival, C55-*P* was dried and resuspended in chloroform:methanol (2:1) at a final concentration of 1 mM. n-dodecyl-beta-D-maltopyranoside (DDM) was purchased from Anatrace (Maumee, USA). All other chemicals were from Millipore Sigma (Gillingham, UK).

### Bacterial strains and plasmids

Bacterial strains and plasmids used in this work are listed in Supplementary Table 1.

### Cloning of overexpression plasmids

DNA encoding for BacA, PgpB and UppS was amplified by PCR from genomic DNA of *E. coli* BW25113 using the appropriate oligonucleotides (Supplementary Table 2) and cloned into pET28a(+) with the appropriate restriction enzymes (Supplementary Table 2) generating the overexpression plasmids pET28a-bacA, pET28a-pgpB and pET28a-ispU, respectively. pET28a-pgpB-mut was generated from pET28a-pgpB by introducing a point mutation using the QuikChange Lightning Kit (Agilent) and the oligonucleotides listed in Supplementary Table 2 and used for the overproduction of the inactive version PgpB(D211E).

### Purification of proteins

#### Purification of PgpB and PgpB(D211E)

PgpB and the inactive PgpB(D211E) version were purified according to (Touzé et al., 2008a) with modifications. PgpB was overproduced in *E. coli* C43(DE3) cells grown in 2YT medium at 37 °C to an OD_600_ of 0.8. Overproduction was induced by addition of 1 mM IPTG (final concentration). After 3.5 h of incubation at 37 °C cells were harvested by centrifugation for 15 min at 7,000*g* and 14 °C. The cell pellet was resuspended in 40 mL of buffer P1 (20 mM Tris/HCl pH 7.5, 500 mM NaCl, 1 mM MgCl_2_, 20 mM β-mercaptoethanol, 10% glycerol) supplemented with 1 mM PMSF, 1 × protease inhibitor cocktail (PIC) and deoxyribonuclease I (Sigma Aldrich). Cells were broken by sonication and centrifuged for 1 h at 130,000 × *g* and 4 °C. The membrane fraction containing pellet was resuspended in 40 mL buffer P2 (20 mM Tris/HCl pH 7.5, 500 mM NaCl, 1 mM MgCl_2_, 10 mM β-mercaptoethanol, 10% glycerol, 1% DDM) supplemented with 1 mM PMSF and 1× PIC and stirred overnight at 4 °C. Insoluble material was removed by centrifugation for 1 h at 130,000*g* at 4 °C. The supernatant was recovered, mixed with 2 mL Ni-NTA Superflow (Qiagen) preequilibrated in buffer P3 (20 mM Tris/HCl pH 7.5, 500 mM NaCl, 1 mM MgCl_2_, 10 mM β-mercaptoethanol, 10% glycerol, 0.05% DDM) supplemented with 10 mM imidazole and gently stirred overnight at 4 °C. Ni-NTA agarose was poured in a gravity flow column and washed 10 times with 8 mL of buffer P3 supplemented with imidazole (5 times with 10 mM and 5 times with 30 mM). PgpB was eluted with buffer C supplemented with 400 mM imidazole. Eluted protein was dialysed against 2 L dialysis buffer (20 mM Tris/HCl pH 7.4, 150 mM NaCl, 10 mM β-mercaptoethanol, 10% glycerol). The combined fractions were concentrated using a Vivaspin Turbo 15 ultrafiltration unit (Sartorius, Göttingen, Germany) and stored in aliquots at −80 °C.

#### Purification of BacA

BacA was purified according to (Manat et al., 2015) with modifications. BacA was overproduced in *E. coli* C43(DE3) cells grown in 2YT medium at 37 °C to an OD_600_ 0.7. Overproduction was induced by addition of IPTG to a final concentration of 1 mM and incubation continued overnight at 22 °C. Cells were harvested by centrifugation for 15 min at 7,000 × *g* and 14 °C. The resulting cell pellet was resuspended in 40 mL buffer B1 (25 mM Tris/HCl pH 7.2, 150 mM NaCl, 5 mM β-mercaptoethanol, 10% glycerol) supplemented with 1 mM PMSF, 1× PIC and deoxyribonuclease I (Sigma Aldrich). Cells were broken by sonication and centrifuged for 1 h at 130,000*g* and 4 °C. The pellet was resuspended in 60 mL buffer B2 (25 mM Tris/HCl pH 7.2, 300 mM NaCl, 5 mM β-mercaptoethanol, 20% glycerol, 2% DDM) supplemented with 1 mM PMSF and 1× PIC (as before) and stirred overnight at 4 °C. Insoluble material was removed by centrifugation for 1 h at 130,000*g* at 4 °C. The supernatant was recovered and further purified using immobilized metal affinity chromatography (IMAC) in a 5 mL HisTrap column (GE Lifesciences) equilibrated in buffer B3 (25 mM Tris/HCl pH 7.2, 300 mM NaCl, 5 mM β-mercaptoethanol, 20% glycerol, 0.1% DDM) supplemented with 10 mM imidazole. The column was washed with 5 column volumes (CV) of buffer B3 supplemented with 10 mM imidazole and with 5 CV of buffer B3 supplemented with 50 mM imidazole. BacA was eluted using buffer B3 supplemented with 300 mM imidazole. Fractions containing BacA were combined and dialysed against buffer B4 (25 mM Tris/HCl pH 7.2, 300 mM NaCl, 5 mM β-mercaptoethanol, 10% glycerol). Optionally, the oligohistidine tag was removed by addition of 2.5 units/mL of thrombin (Novagen) while dialysing in buffer B4 for 20 h. The protein was further purified using gel filtration chromatography on a Superdex 200 HiLoad 16/60 column (GE Healthcare) equilibrated in buffer B3 at 1 mL/min. Fractions were analysed using SDS-PAGE and fractions containing BacA were combined, concentrated using a Vivaspin Turbo 15 ultrafiltration unit (Sartorius, Göttingen, Germany) and stored in aliquots at −80 °C.

#### Purification of UppS

UppS was purified according to (Pan et al., 2000) with modifications. UppS was overproduced in *E. coli* BL21(DE3) (Novagen) cells grown in LB medium at 37 °C to an OD_600_ of 0.6. Overproduction was induced by addition of IPTG to a final concentration of 1 mM. After 3.5 h of incubation at 37 °C cells were harvested by centrifugation for 15 min at 7,000*g* and 14 °C. The resulting cell pellet was resuspended in 80 mL buffer (25 mM Tris/HCl pH 7.8, 150 mM NaCl) supplemented with 1 mM PMSF, 1× PIC and deoxyribonuclease I. Cells were broken by sonication and centrifuged for 1 h at 130,000*g* and 4 °C. The supernatant was mixed with 2 mL Ni-NTA Superflow preequilibrated in buffer (supplemented with 5 mM imidazole) and gently stirred at 4 °C for 2–3 h. Ni-NTA agarose was poured in a gravity flow column, washed 5 times with 10 column volumes of buffer supplemented with 30 mM imidazole. UppS was eluted with buffer supplemented with 300 mM imidazole. Eluted protein was dialysed against 2 L buffer and further purified by size exclusion chromatography on a Superdex 200 HiLoad 16/60 column (GE Healthcare) using the same buffer and a flowrate of 1 mL/min. Fractions containing UppS were combined, concentrated using a Vivaspin Turbo 15 ultrafiltration unit (Sartorius, Göttingen, Germany) and stored in aliquots at −80 °C.

#### Other proteins

PBP1B (Bertsche et al., 2006, Typas et al., 2010), PBP1A (Born et al., 2006) and LpoB (Egan et al., 2014) were purified as described.

### Cefsulodin susceptibility assays

Disc diffusion assays were carried out as described by the European Committee on Antimicrobial Susceptibility Testing (EUCAST) with modifications (EUCAST, 2017). Briefly, 10 mL fresh Mueller-Hinton broth was inoculated with material from one bacterial colony and grown until the OD_600_ reached 0.5. The cell suspension was diluted to an OD_600_ of 0.125, 1 mL was centrifuged for 1 min at 3500*g* and resuspended in 1 mL of 0.9% NaCl. Bacteria were evenly distributed onto a fresh Mueller-Hinton II agar plate with 4 mm agar thickness using a cotton swab soaked with the cell suspension. The plate was allowed to dry for 5 min and a paper disc containing 30 µg cefsulodin was placed in the centre. Plates were incubated for 18 h at 37 °C. A high resolution picture was taken for each plate and the diameter of the growth inhibition zone was determined using the program ImageJ.

### Cell viability assay (spot-plate-assay)

Overnight cultures of the test strains grown in Mueller-Hinton broth were diluted to an OD_600_ of 2. A serial dilution series (10^0^ to 10^−7^) was prepared in Mueller-Hinton broth using a 96-well plate. Cells from each dilution step were applied on Mueller-Hinton II agar plates containing 32 µg/mL cefsulodin using a replica plater (Sigma Aldrich), dried and incubated for 18 h at 37 °C. For growth control Mueller-Hinton II agar plates without antibiotic were used.

### *In vitro* pull down assay with or without cross-linking

Proteins were mixed in 200 μL of binding buffer (10 mM HEPES/NaOH, 10 mM MgCl_2_, 300 mM NaCl, 0.1% Triton X-100, pH 7.5). The protein concentrations were 1.5 or 2.0 μM for PBP1A or PBP1B, 3 μM for PgpB-ht and 5 μM for ht-BacA. Samples were incubated at ambient temperature for 10 min to allow possible complexes to form. If cross-linking was required, 0.2% (w/v) formaldehyde (Millipore Sigma) was added followed by incubation at 37 °C for 10 min. Excess cross-linking was blocked by addition of 100 mM Tris/HCl, pH 7.5. Complexes were pulled down by O/N incubation at 4 °C with 100 µL of washed and equilibrated Ni-NTA superflow beads (QIAGEN, Hilden, Germany). Beads were washed 5–8 times with 1.5 mL of wash buffer (10 mM HEPES/NaOH, 10 mM MgCl_2_, 500 mM NaCl, 50 mM imidazole, 0.05% Triton X-100, pH 7.5). For samples without cross-linker a lower imidazole concentration of 20 mM was used in the wash buffer. Retained proteins were eluted by boiling the beads in SDS-PAGE loading buffer; beads were then removed, and samples resolved by SDS-PAGE. Gels were stained with Coomassie brilliant blue (Carl Roth, Germany).

### Reconstitution of PBP1B, PgpB and BacA in liposomes

Proteoliposomes containing PBP1B in the presence or absence of BacA or PgpB were prepared as described previously with some modifications (Egan et al., 2015, Rigaud and Lévy, 2003). The appropriate lipid or mixture of lipids were dried in a glass test tube under stream of N_2_ to form a lipid film followed by desiccation under vacuum from 2 h. Resuspension into multilamellar vesicles (MLVs) was achieved by addition of the indicated buffer (see below) and several cycles of vigorous mixing and short incubations in hot tap water. *E. coli* polar lipids were resuspended in 20 mM Tris/HCl pH 7.5 while 20 mM Tris/HCl pH 7.5, 150 mM NaCl was used for artificial lipids. The final lipid concentration was 5 g/L. To form large unilamellar vesicles (LUVs), MLVs were subjected to 10 freeze-thaw cycles and then extruded 10 times through a 0.2 µm filter. LUVs were destabilised by the addition of Triton X-100 to an effective detergent:lipid ratio of 1.40 and mixed with proteins in a 1:3000 lipid:PBP1B molar ratio and 1:1 PBP1B:phosphatase molar ratio. After incubation at 4 °C for 1 h, prewashed Biobeads SM2 (BioRad, USA; 100 mg per 3 µmol of Triton X-100) were added to the sample to remove detergents. Biobeads were exchanged after 2 and 16 h, followed by incubation with fresh Biobeads for a further 2 h. After removal of Biobeads by short centrifugation at 4,000*g*, liposomes were pelleted at 250,000*g* for 30 min at 4 °C. The pellet containing proteoliposomes was resuspended in the appropriate buffer and analysed by SDS-PAGE and a bicinchoninic acid assay (Pierce BCA Assay Kit, ThemoFisher Scientific, USA) to determine protein concentration. The size of LUVs and proteoliposomes was confirmed using dynamic light scattering (DLS) using a Zetasizer instrument (Malvern Technologies, UK).

### Phosphatase assay for PgpB and BacA

Phosphatase assays were carried out as previously described (Touzé et al., 2008a). Assays were performed with a total volume of 50 µl in buffer containing 20 mM Tris/HCl pH 7.5, 150 mM NaCl, 10 mM β-mercaptoethanol, 0.02% DDM, and 10 mM MgCl_2_. The substrate farnesyl pyrophosphate (Millipore Sigma) was added at a range of concentrations from 5 to 80 µM. The enzyme concentrations were 42 nM PgpB, 42 nM BacA, and 66 nM PBP1B. After incubation for 15 min at ambient temperature 140 µL Biomol Green reagent (Enzo Life Sciences, BML-AK111-0250) was added and samples were incubated for 30 min at ambient temperature to allow for colour development. Absorbance at 620 nm was measured in a SpectraMax M2 spectrophotometer. Inorganic phosphate standard provided with the Biomol Green reagent was used to obtain a standard curve that was used to convert enzyme assay absorbance into phosphate concentration. The enzyme kinetics curves were analysed using classical Michaelis–Menten equation using the Excel add-on, Solver.

### C55-*PP* synthesis by UppS

C55-*PP* synthesis reaction was carried out as described in (Pan et al., 2000) with minor modifications. The reaction contained 50 mM HEPES/KOH pH 7.5, 50 mM KCl, 0.5 mM MgCl_2_, 0.1% Triton X-100, 2 nmol C15-*PP*, 22 nmol C5-*PP* and 10 µM UppS. Each reaction was incubated for 4 h at 25 °C under continuous shaking (600 rpm).

### Continuous glycosyltransferase (GTase) assay

Continuous fluorescence GTase assays using dansylated lipid II were performed as published (Banzhaf et al., 2012, Egan and Vollmer, 2016). Samples contained 50 mM HEPES/NaOH pH 7.5, 150 mM NaCl, 10 mM MgCl_2_ 0.04% Triton X-100 and 0.01% DDM (reactions with BacA) or 0.006% DDM (reactions with PgpB) in a final volume of 60 µL. PBP1B and phosphatases were added at a concentration of 0.5 µM and 1 µM, respectively. When indicated, LpoB(sol) was added at a concentration of 2 µM. Reactions were started by the addition of dansylated lipid II to a final concentration of 10 µM and monitored by following the decrease in fluorescence over 30 min at 37 °C using a FLUOstar OPTIMA plate reader (BMG Labtech, Germany) with excitation at 330 nm and emission at 520 nm. The fold-increase in GTase rate was calculated against the mean rate obtained with PBP1B alone at the same reaction conditions, at the fastest rate.

The assay was modified to test the effects of C55-*P* or C55-*PP* on GTase activity. First, reactions to synthesise C55-*PP* with UppS and the substrates C5-*PP* and C15-*PP* were carried out as described above. Then these samples were mixed with PBP1B, LpoB in the presence or absence of PgpB or PgpB(D211E). Control reactions were performed in which either only the substrates, only UppS or the UppS buffer were added. Samples had a final volume of 60 µL and contained 50 mM HEPES/KOH pH 7.5, 115 mM KCl, 35 mM NaCl, 0.5 mM MgCl_2_, 1.2 mM β-mercaptoethanol, 1.3% glycerol, 0.035% Triton X-100, 0.006% DDM, 50 µM moenomycin (only for negative controls), 3.09 µM UppS, 0.14 mg/mL cellosyl, 1 µM PgpB or PgpB(D211E), 0.5 µM PBP1B and 1 µM LpoB. Sample further contained either 33.3 µM C55-*PP* (from reactions with UppS and substrates), 33.3 µM C15-*PP* and 333.3 µM C5-*PP* (mock reactions with substrates) or 33.3 µM C55-*P*. Reactions were started by adding dansylated lipid II to a final concentration of 10 µM and monitored by following the decrease in fluorescence over 60 min at 25 °C using a FLUOstar OPTIMA plate reader as described above.

### *In vitro* peptidoglycan synthesis assay using radiolabelled lipid II

To assay the *in vitro* PG synthesis activity of PBP1B with radiolabelled lipid II substrate in the presence of detergent we used a previously published assay (Banzhaf et al., 2012, Biboy et al., 2013). Final reactions included 10 mM HEPES/NaOH pH 7.5, 150 mM NaCl, 10 mM MgCl_2_ 0.05% Triton X-100 and 0.027% DDM. The concentration of PBP1B was 0.5 or 0.05 µM, and BacA and PgpB were added at a concentration of 2 µM. Reactions were carried out for 1 h at 37 °C. The same methodology with minor modifications was used to assay the *in vitro* PG synthesis activity of PBP1B in liposomes. To start reactions, 1.5 nmol [^14^C]-labelled lipid II were dried in a 0.5 mL glass tube using a vacuum concentrator, resuspended in 5 µL of the appropriate liposome buffer, and mixed with liposomes, buffer and MgCl_2_ to a total volume of 50 µL. Final reactions contained 0.5 µM PBP1B, 30 µM lipid II and 1 mM MgCl_2_ in 20 mM Tris/HCl pH 7.5. Samples were incubated for 90 min at 37 °C with shaking at 800 rpm. Reactions were stopped by boiling for 5 min. Digestion with cellosyl, reduction with sodium borohydride and analysis by HPLC were performed as described (Biboy et al., 2013).

### Coupled PG synthesis/C55-*PP* phosphatase assay in liposomes

Phosphatase assays were carried out in a final volume of 50 µl containing 10 mM Tris/HCl pH 7.5, 150 mM NaCl, 1 mM MgCl_2_, 0.5 mM CaCl_2_, 40 µM lipid II, 0.95 µM protein (protein content in liposomes), 1 µM LpoB(sol) and 2 nmol lipid II. The mixture was incubated for 4 h at 37 °C. Reactions were terminated by adding 50 µl n-butanol/pyridine acetate pH 4.2 (2:1). Samples were vortexed for 1 min and centrifuged for 3 min at 17,000*g* on a bench top centrifuge. The reaction products present in the organic phase were analysed by TLC on HPTLC alumina silica gel 60 plates (Merck KgaA) using chloroform-methanol-water-ammonia (88:48:10:1) as the mobile phase (Rick et al., 1998). TLC bands were stained with iodine and quantified using ImageJ software, the data were analysed using Excel.

### Surface plasmon resonance (SPR) experiments

PBP1A and PBP1B were immobilised covalently on an SPR sensorchips as previously described (Egan et al., 2014). PgpB was injected in 10 mM Tris/maleate pH 7.5, 150 mM NaCl, 5 mM imidazole, 5 mM β-mercaptoethanol, 0.05% DDM; BacA was injected in 10 mM Tris/maleate at pH 7.5, 300 mM NaCl, 0.1% Triton X-100. The concentration of PgpB injected ranged from 0.125–2 μM. BacA was injected at a concentration range of 0.163–3 μM. Assays were performed in triplicate at 25 °C and a flow rate of 75 μL/min, and with an injection time of 5 min. Binding curves were obtained by plotting the equilibrated response signal at the end of the injection minus the signal from the channel without immobilized protein, against ligand concentration. The resulting binding curves were fitted using the non-linear least squares method to a model for 1:1 binding described by Eq. (1) in which *R* is the SPR signal at the equilibrium, *R_max_* the maximum response, *K_D_* the dissociation constant and C the analyte concentration.

(1) $$\text{R}=\frac{R_{\text{max}}\cdot \text{C}}{K_{D}\text{+C}}$$

The non-linear regression was performed using SigmaPlot 13 software (Systat Software Inc.).

### Structural model for the PgpB-PBP1B complex

The docking models of PgpB-PBP1B complex were built using HADDOCK2.2 data-driven docking protocols (Dominguez et al., 2003) and CNS1.2 (Brünger et al., 1998) for the structure calculations. The initial coordinates of *E. coli* PgpB (PDB code 5JWY (Tong et al., 2016)) and *E. coli* PBP1B (PDB code 5HLD (King et al., 2017)) were used and the docking energy minimization occurred with a minimal conformational rearrangement of the partners. The multi domain docking was driven with ambiguous interaction restraints between the transmembrane helix of PBP1B (active residues 74–96) and PgpB (passive residues 1–235). The HADDOCK score was used to rank the generated models (Lutje Hulsik et al., 2013).

## Results

### *PgpB* deletion affects the function of PBP1B in the cell

**Fig. 2:**
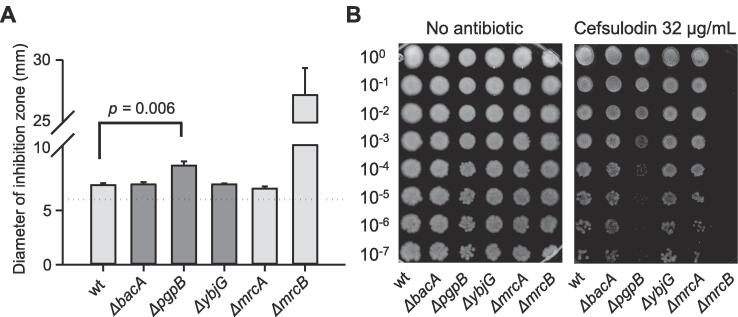
A Δ*pgpB* mutant shows increased susceptibility to cefsulodin. (A) The growth inhibition zone around discs loaded with 30 µg of cefsulodin was significantly increased in the mutant strain lacking PgpB compared to wt strain (BW25113) or strains lacking BacA, YbgG or PBP1A (Δ*mrcA*). As expected, the mutant lacking PBP1B (Δ*mrcB*) was hyper-susceptible to cefsulodin. The data are mean ± SD of three independent experiments. (B) Spot plate assay showing the increased susceptibility of the Δ*pgpB* mutant on plates containing 32 µg/mL cefsulodin.

The β-lactam cefsulodin targets PBP1A from *E. coli* with high affinity and an IC50 of 0.47 μg/mL. It also inhibits the homologous PBP1B with an IC50 of 3.7 μg/mL but does not target any other PBP in *E. coli* at a workable concentration (Curtis et al., 1979). Because the cell needs either PBP1A or PBP1B for growth, any condition or mutation that affects PBP1B function causes a higher susceptibility for cefsulodin. When testing the cefsulodin susceptibility of different *E. coli* mutants either by measuring the inhibition zone in a disc diffusion assay (Fig. 2A) or by a spot plate based cell viability assay (Fig. 2B) we first confirmed that a Δ*mrcB* mutant (lacking PBP1B), but not a Δ*mrcA* mutant (lacking PBP1A) showed a higher susceptibility compared to *E. coli* wild-type, indicating that indeed PBP1B becomes more important in cells growing in the presence of cefsulodin (Sarkar et al., 2012). Moreover, we noticed that a mutant lacking the C55-*PP* pyrophosphatase PgpB displayed increased sensitivity to cefsulodin (Fig. 2). Mutants lacking BacA or YbjG had similar sensitivities to cefsulodin as the wild-type (Fig. 2). These data suggest that the functionality of PBP1B might be reduced in the cell when PgpB is absent.

### PgpB interacts with PBP1B

**Fig. 3:**
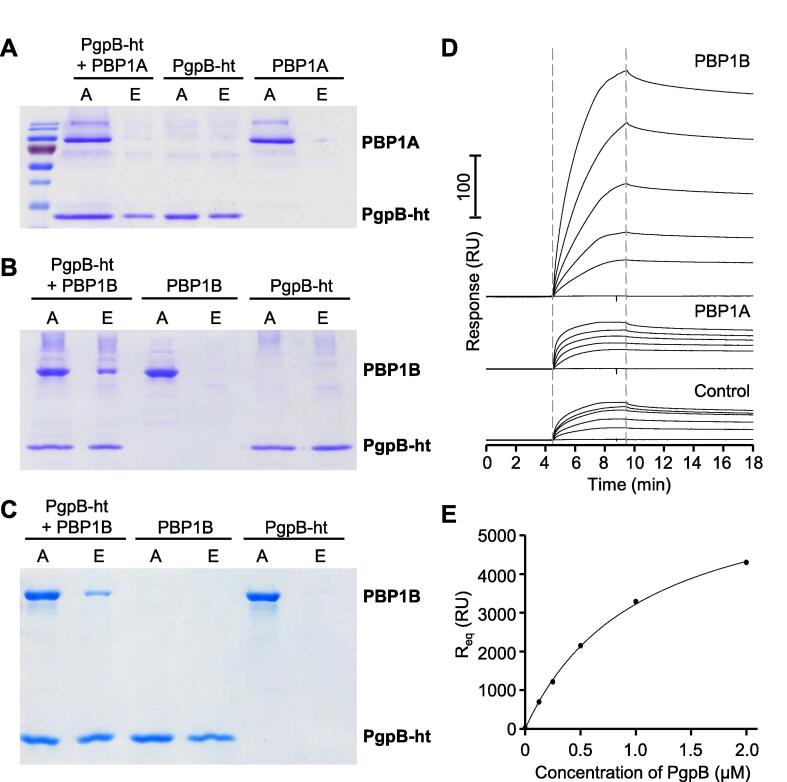
PgpB interacts with PBP1B but not with PBP1A. (A) Oligohistidine tagged PgpB (PgpB-ht) and PBP1A alone, or both proteins together were incubated with chemical cross-linker and followed by pull-down to Ni-NTA beads. PgpB-ht did not interact with PBP1A. PgpB-ht did pull down PBP1B to Ni-NTA beads in the presence (B) or absence (C) of chemical cross-linker. Lanes labelled with A correspond to samples applied to the Ni-NTA beads and E to the elution. (D) PBP1A, PBP1B or no protein were immobilized on a sensor chip with bound ampicillin. PgpB-ht was applied at concentrations of 2, 1, 0.5, 0.25 or 0.125 µM. PgpB-ht bound to immobilised PBP1B but not to PBP1A or the control surface. (E) Analysis of the titration data in panel D using a 1:1 binding model yields an apparent K_D_ of 1.05 ± 0.06 µM (three independent experiments).

The phenotypic analysis of the Δ*pgpB* mutant and the proximate position of PgpB and PBP1B in the PG synthesis pathway - the product of PBP1B is the substrate of PgpB - pointed to a possible interaction between both proteins. To test this possibility we purified both proteins, the second major bi-functional PG synthase, PBP1A and the major C55-*PP* phosphatase BacA, for protein-protein interaction studies. A C-terminal His_6_-tagged version of PgpB (Touzé et al., 2008a), PgpB-ht, was used in pull-down assays with non-tagged PBP1A or PBP1B initially in the presence of the chemical cross-linker formaldehyde, followed by incubation with Ni-NTA beads to capture PgpB-ht along with any proteins associated with it. PgpB-ht was able to pull down untagged PBP1B but not untagged PBP1A (Fig. 3A and B). Both PBPs were not pulled down by the beads in the absence of PgpB-ht. PgpB-ht also pulled down untagged PBP1B in the absence of the cross-linker (Fig. 3C), suggesting that both proteins interact directly.

To verify the specific interaction between PgpB and PBP1B, we used surface plasmon resonance (SPR) experiments in which PBP1A or PBP1B were covalently immobilized via their TPase active site to surfaces with bound ampicillin (Fig. 3D). PgpB bound strongly to a surface containing PBP1B, while the binding of PgpB to a control surface without protein or a surface with immobilized PBP1A was significantly weaker. This indicated a specific interaction between PgpB and PBP1B but not with PBP1A, in agreement with our pull down assays. The binding of PgpB to surfaces with immobilized PBP1B increased in a concentration-dependent manner. These binding responses could be fit to a 1:1 binding model yielding an apparent dissociation constant of 1.05 ± 0.06 µM (Fig. 3E).

In addition to PgpB, we also tested BacA for possible interactions with PBP1A or PBP1B (Fig. S1). An N-terminal His_6_-tagged version, ht-BacA, was purified according to published protocols (Manat et al., 2015) and used in pull down assays with non-tagged PBP1B in the presence or absence of the cross-linker formaldehyde (Fig. S1A and B). ht-BacA was able to pull down PBP1B independently of the addition of cross-linker, indicating an interaction between both proteins. In order to further characterize this interaction, we performed SPR experiments, injecting BacA onto surfaces containing covalently immobilised PBP1A or PBP1B, as done before for PgpB (Fig. S1C). We detected a slightly higher binding signal when BacA was injected onto the PBP1B surface compared to the signal obtained for the PBP1A-containing surface or the control surface without protein (Fig. S1D and E). However, BacA bound significantly to the control surface (dextran with immobilized and hydrolysed ampicillin) and we could not reach a sufficiently high concentration of BacA to saturate the binding to the PBP1B surface, preventing calculation of a binding constant (Fig. S1E).

### PgpB and BacA stimulate PBP1B activity in detergents

The physical interactions between C55-*PP* phosphatases and PBP1B could allow the successive reactions of both enzymes to be coupled, affecting the reaction rates. Therefore we tested first whether the presence of PBP1B affects the phosphatase activity of PgpB or BacA, and second whether the presence of BacA or PgpB affected PG synthesis by PBP1B.

**Fig. 4:**
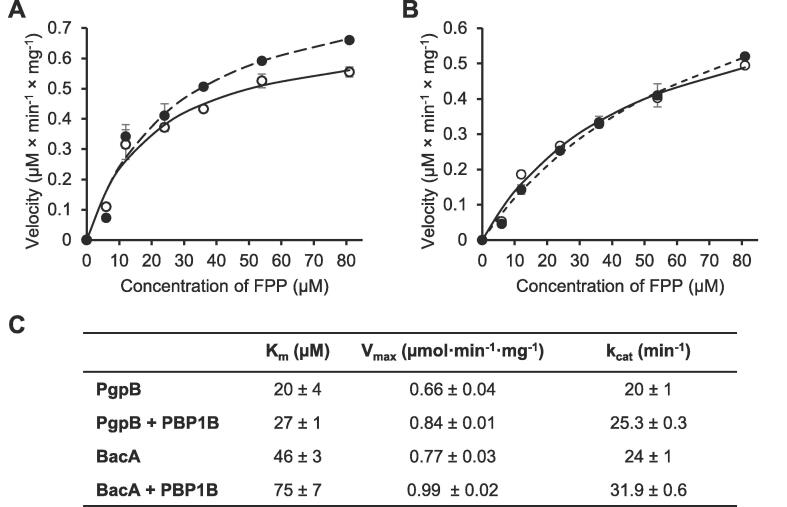
PBP1B has a mild effect on the phosphatase activities of PgpB and BacA. (A) Activity of PgpB against in farnesyl pyrophosphate the presence or absence of PBP1B. (B) Activity of BacA farnesyl pyrophosphate in the presence or absence of PBP1B. In both (A) and (B), white symbols correspond to the phosphatase on its own and black symbols to the phosphatase plus PBP1B. (C) Kinetic parameters (mean ± SD) obtained in three independent experiments.

We measured the activities of PgpB or BacA in the presence or absence of PBP1B, using farnesyl pyrophosphate (C15-*PP*) as substrate (Fig. 4A and B). The presence of PBP1B had only small effects, slightly increasing the K_m_ and k_cat_ values. The strongest effect was on BacA activity, which had a K_m_ of 20 ± 4 µM without PBP1B and 46 ± 3 µM with PBP1B. The increase in k_cat_ in the presence of PBP1B was ∼1.3-fold for both phosphatases (Fig. 4C). PBP1B did not have phosphatase activity on its own (data not shown).

**Fig. 5:**
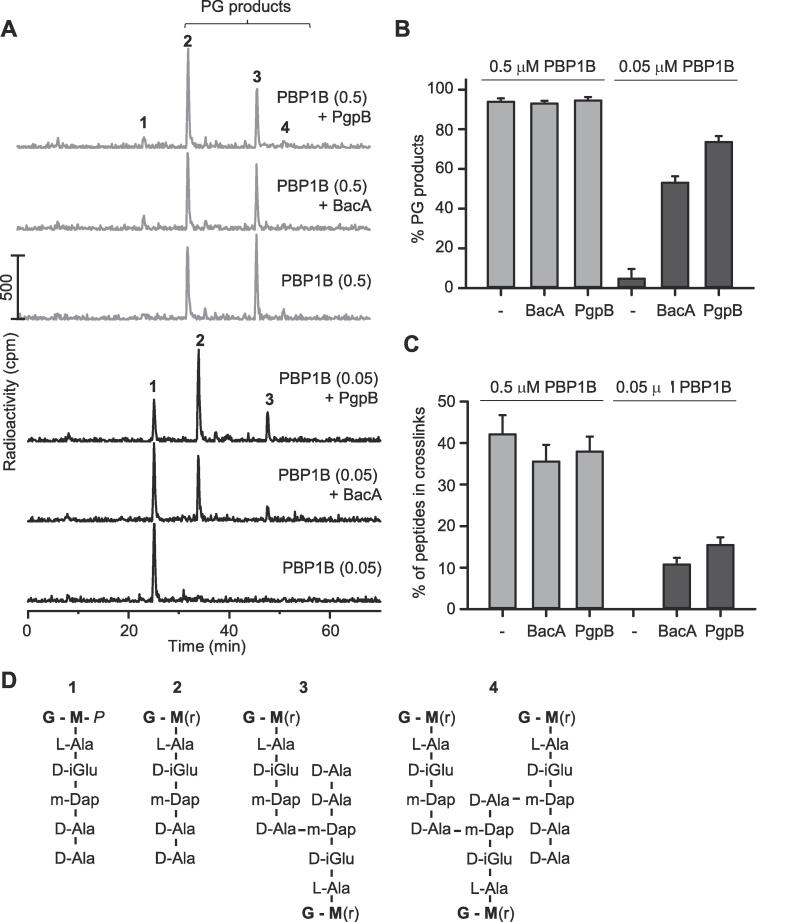
PgpB and BacA stimulate PBP1B at low concentration in the presence of Triton X-100. (A) PBP1B was incubated with radiolabelled lipid II in the presence or absence of BacA or PgpB. The reaction was stopped by boiling. The PG was digested with the muramidase cellosyl, reduced with sodium borohydride, and the resulting muropeptides were separated by HPLC. The concentration of PBP1B in µM is indicated in brackets. (B) Quantification of the PG products obtained in at least three independent experiments (mean ± SD). (C) Quantification of the % peptides in cross-links obtained in at least three independent experiments (mean ± SD). (D) Proposed structures of muropeptides in fractions 1–4 (panel A).

We next performed *in vitro* PG synthesis assays to assess the effect of phosphatases on the GTase and TPase activities of PBP1B (Figs. 5 and S2). The continuous GTase assay uses fluorescently labelled lipid II (dansyl-lipid II) and the PG polymers produced are digested to muropeptides with reduced fluorescence. We assayed PBP1B at two concentrations (0.5 and 0.05 µM, respectively) with or without its activator LpoB and in the presence or absence of PgpB and BacA (Fig. S2). PBP1B has poor activity at the lower concentration (Müller et al., 2007) and by testing this condition we would detect a potentially stimulatory effect. As shown before (Egan et al., 2014), PBP1B had a significant higher relative GTase rate in the presence of LpoB. However, neither BacA nor PgpB affected the GTase rate of PBP1B at any of the conditions tested (Fig. S2C and F).

We then used an end point assay with radiolabelled lipid II to test the coupled GTase and TPase activities of PBP1B. The PG produced was digested with the muramidase cellosyl and the resulting muropeptides we separated by high-pressure liquid chromatography (HPLC) (Fig. 5). This assay does not measure enzyme kinetics but has the advantage that both activities of PBP1B can be monitored in the same sample with the chemically unaltered substrate. As before, we assayed PBP1B at concentrations of 0.5 and 0.05 µM, respectively, and in the presence or absence of each phosphatase (Fig. 5A). At the high concentration of PBP1B, PgpB and BacA did not affect the amount of PG produced (Fig. 5B) and did not significantly affect the amount of TPase products, the cross-linked muropeptides (Fig. 5C). At the low concentration of PBP1B, PgpB and BacA stimulated the consumption of lipid II and the amount of cross-linked muropeptides (Fig. 5B and C). Moreover, the stimulatory effect of PgpB was higher than that of BacA (Fig. 5B and C).

In summary, we detected a small but significant stimulatory effect of PBP1B on BacA and PgpB, and an activation of PBP1B at low concentration by both phosphatases.

### PgpB and BacA stimulate PBP1B in liposomes

The enzymatic assays described above were carried out in the presence of detergents with the solubilized membrane enzymes. However, detergents may affect the activity of membrane proteins and the diffusion of substrates and proteins in a membrane system is different from the three-dimensional diffusion occurring in detergents, which could hide or distort the effects of the coupling of the two reactions. To measure the activities of PBP1B in a more physiological environment we reconstituted it in liposomes in the presence or absence of BacA or PgpB. We prepared these liposomes with either *E. coli* polar lipids (EcPL), an extract of natural lipids from *E. coli*, or DOPC:POPG:TOCL 70:20:10 (molar ratio), a mixture of artificial lipids matching the contents of lipids in EcPL except for the substitution of phophatidylethanolamine with phosphatidylcholine to improve liposome stability. We found that the activity of PBP1B was higher in liposomes prepared from the natural lipid extract than the artificial lipids (Fig. S3) and used these in most activity assays. Liposomes made of artificial lipids were only used when assaying phosphatase activity because we realized that EcPL contains a significant amount of C55-*P* that can be detected in the thin layer chromatography (TLC) assays (data not shown).

**Fig. 6:**
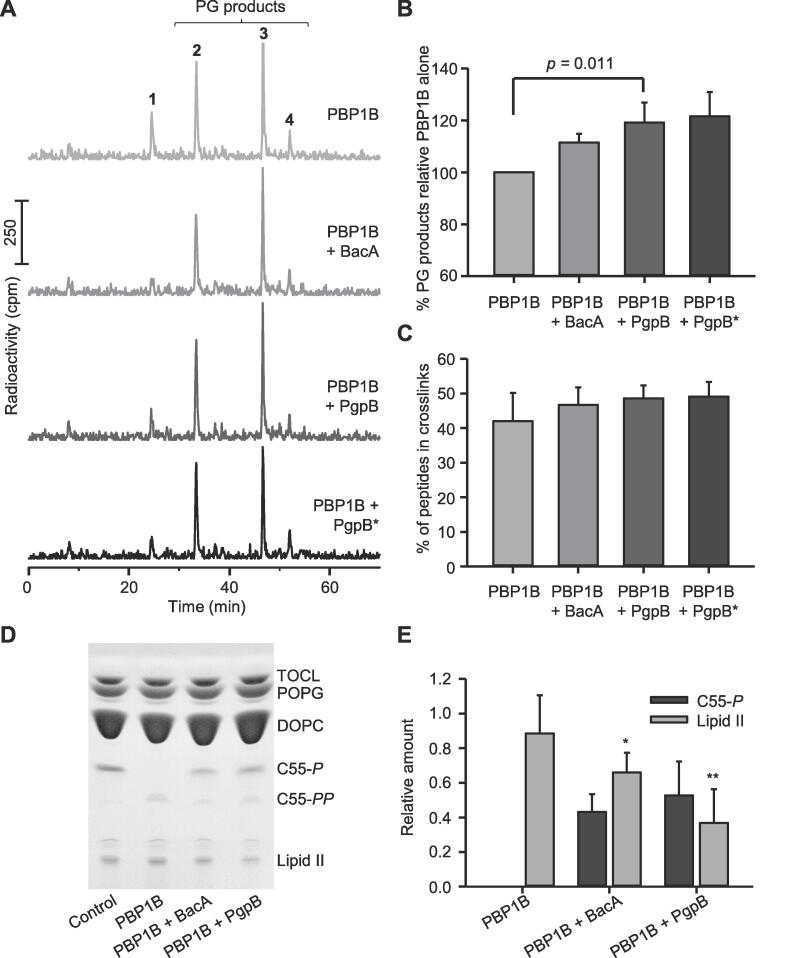
BacA and PgpB increase lipid II consumption by PBP1B in the membrane. The activity of PBP1B was measured in proteoliposomes with PgpB, BacA or no phosphatase. (A) Radiolabelled lipid II was used as substrate and PG products were analysed by HPLC (as shown in Fig. 5A). The peak numbers relate to the muropeptide structures in Fig. 5D. (B) Quantification of the PG products obtained in two independent experiments (mean ± SD). (C) Quantification of the % peptides in cross-links obtained in two independent experiments (mean ± SD). (D) Using non-labelled lipid II and thin-layer chromatography allowed the detection of residual lipid II, C55-*P* and C55-*PP*. Control, proteoliposomes without enzyme with added lipid II and C55-*P*. (E) Quantification of lipid II and C55-*P* from 3 independent experiments as the one in panel D. The values are mean ± SD and asterisks indicate *p*-value comparisons with PBP1B (^*^p < 0.1 and ^**^p < 0.05). HPLC assays were performed using *E. coli* polar lipids liposomes. For the TLC assay, artificial lipids were used plus LpoB(sol) was added at 4 µM.

We reconstituted PBP1B in EcPL liposomes either alone or in combination with BacA or PgpB at a molar ratio of 1:1. After reconstitution, we assayed PBP1B activity by the addition of radioactively-labelled lipid II, which is incorporated into the bilayer and consumed by PBP1B. The products of the reaction were digested with cellosyl and analysed by HPLC (Fig. 6A). The chromatograms revealed an increase in the amount of PG produced by PBP1B when phosphatases were present (Fig. 6B). Remarkably, this stimulatory effect was also seen in the presence of the inactive PgpB(D211E) mutant in the liposomes (Fig. 6B). Finally, there was no significant effect on the cross-linkage of the PG product due to the presence of phosphatases (Fig. 6C).

Next, we assayed coupled PG synthesis and phosphatase activities on liposomes. PBP1B was reconstituted into liposomes made of artificial lipids as described above, in the presence or absence of BacA or PgpB at a molar ratio of 1:1. Due to the low activity of PBP1B in the presence of artificial lipids we added a soluble version of its cognate activator, LpoB(sol). The PG synthesis reaction was started by adding lipid II. After the reaction all lipids were extracted with n-butanol/pyridine acetate and analysed by TLC, which also contained lanes with C55-*P* and lipid II standards, and lipid extract from protein-free liposomes (Fig. 6D). Lipid II consumption was observed as the disappearance of the lipid II band in comparison to the standard. We also quantified the band corresponding to C55-*P* produced by the phosphatases but were unable to precisely quantify C55-*PP* due to a buffer component migrating to a similar position on the TLC plate. Liposomes carrying only PBP1B consumed less than 20% of the total lipid II, and the band corresponding to the C55-*PP* was also visible (Fig. 6D and E). When liposomes contained both, PBP1B and a phosphatases the lipid II consumption increased to 34% (with BacA) or 63% (with PgpB), and the band corresponding to C55-*P* appeared in the TLC (Fig. 6D and E). In addition, this assay showed that BacA and PgpB were able to use the C55-*PP* released by PBP1B during PG synthesis. Finally, the stimulatory effect of PgpB was higher than that of BacA in both membrane-based assays (Fig. 6) and in the HPLC assay in detergents (Fig. 5).

### C55-*PP* is a potent inhibitor of PBP1B

**Fig. 7:**
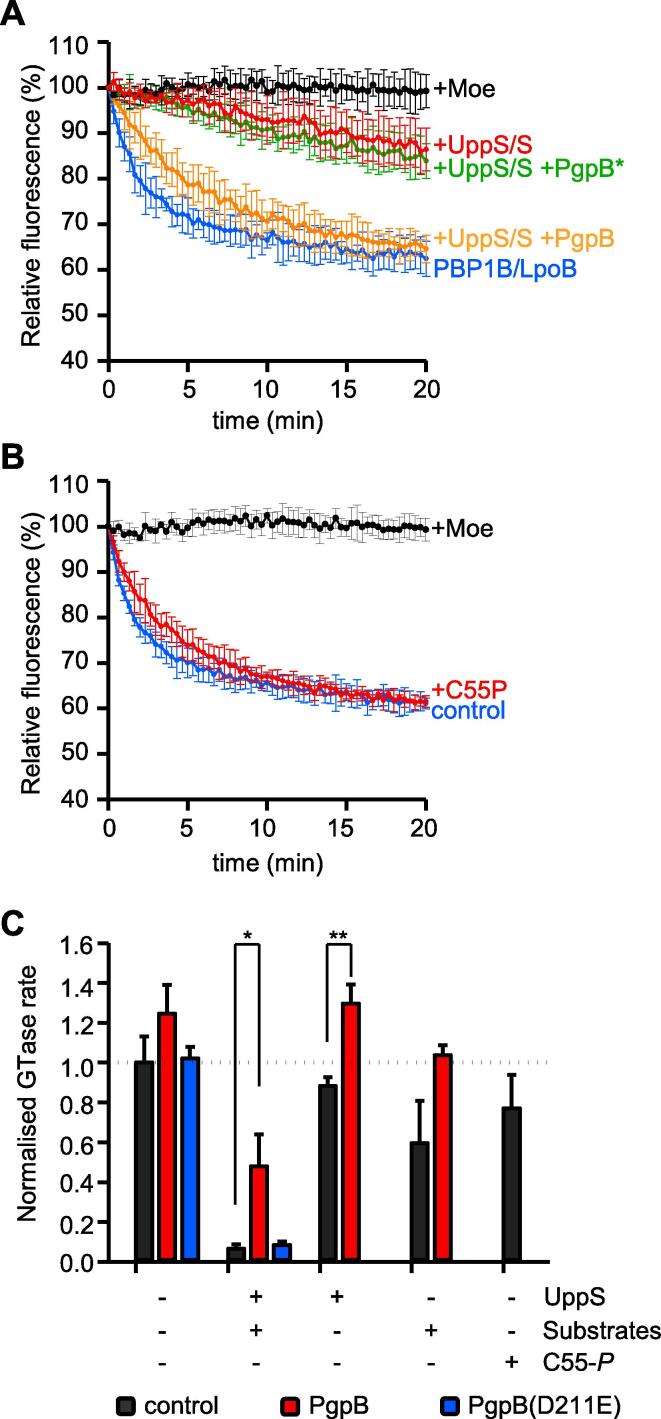
C55-*PP* inhibits the GTase activity of PBP1B. (A) Continuous fluorescence assay for GTase activity of PBP1B in the presence of C55-PP synthesized by incubating UppS and its substrates C5-*PP* and C15-*PP* (+UppS/S). Samples contained PgoB, PgpB(D211E) (PgpB*) or no phosphatase; control samples contained only UppS or substrates, or the known GTase inhibitor moenomycin (Moe). (B) GTase activity assay of PBP1B in the presence or absence of C55-*P.* (C) The GTase rates obtained from the assays in (A) and (B) and control reactions in Fig. S4B–D were normalised relative to the activity of PBP1B in the absence of PgpB, UppS and its substrates. ^*^p < 0.05; ^**^p < 0.005. The values are mean ± SD of at least 3 independent experiments.

To better understand the mechanism by which C55-*PP* pyrophosphatases stimulate PG synthesis we next investigated whether any of the carrier lipid versions had an effect on the GTase activity of PBP1B (Fig. 7). Commercially available C55-*P* had a small inhibitory effect on PBP1B, reducing the relative GTase rate by 23% (Fig. 7B and C). C55-*PP* is not commercially available and was therefore synthesized by UppS from the substrates C5-*PP* (isopentenyl pyrophosphate, IPP) and C15-*PP* (farnesyl pyrophosphate, FPP) prior to starting the GTase reaction by addition of dansylated lipid II (Fig. S4A). Interestingly, we found that C55-*PP* is a potent inhibitor of PBP1B, reducing its relative GTase rate by 93% (Fig. 7A and C). The presence of UppS without its substrates had no effect on PBP1B; C5-*P* and C15-*PP* without UppS caused an 40% reduction in PBP1B activity (Fig. 7C and S4B). Importantly, the addition of active PgpB, but not the inactive version, partially restored the GTase activity of PBP1B, presumably by converting some of the C55-*PP* produced by UppS to C55-*P*, which is less inhibitory (Fig. 7A, C, S4C and S4D). Interestingly, PgpB also protected PBP1B from inhibition by UppS substrates C5-*PP* and C15-*PP* most probably by dephosphorylating these (Fig. 7C and S4C). Hence, our data show that PBP1B is inhibited by its product, C55-*PP*, which is released in GTase reactions, and that activity can be restored by dephosphorylation of C55-*PP* to C55-*P*.

### *In silico* modelling of the structure of the PgpB-PBP1B complex

**Fig. 8:**
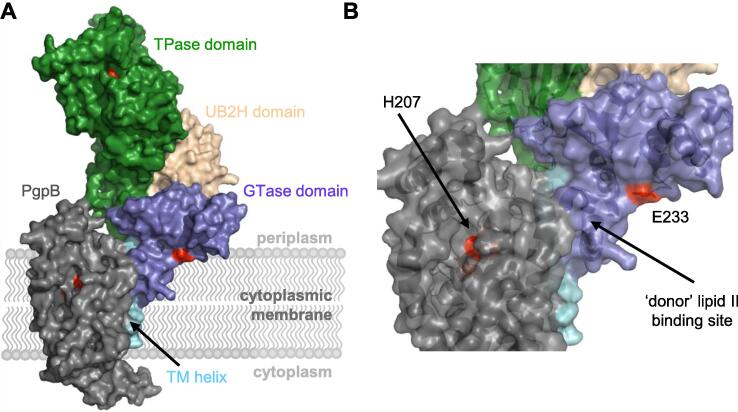
Docking model of the PgpB-PBP1B complex. The complex between PgpB and PBP1B was built and energy-minimised with HADDOCK/CNS programs starting from the initial coordinates of the *E. coli* structures (PDB codes 5JWY and 5HLD). (A) Structure of the lowest energy complex depicted in its surface representation. PgpB is shown in grey, the domains of PBP1B are coloured in blue (GTase), green (TPase), cyan (UB2H), and light blue (trans-membrane helix). Catalytic residues in PgpB (H163, H207, D211) and PBP1B (E233 in the GTase domain and S510 in the TPase domain) are coloured in red. (B) A zoomed-in view of active site regions showing the proximity between active site of PgpB and the ‘donor’ lipid II binding site in the GTase domain of PBP1B.

In order to better understand the interaction of PgpB and PBP1B, we performed an *in silico* docking study using the published crystal structures of PgpB (Tong et al., 2016) and PBP1B (King et al., 2017) (Fig. 8). The complex was built and energy-minimised as described in Materials and Methods. The lowest energy clusters have a HADDOCK scores of −108.3 ± 17.4 kcal mol^−1^ with electrostatic and Van der Waals energies of −94.3 ± 25.8 kcal mol^−1^ and −69.7 ± 4.8 kcal mol^−1^, respectively. In the lowest energy model, the active sites of PgpB and the GTase of PBP1B GTase are both on the periplasmic side of the cytoplasmic membrane, as has been shown experimentally. The single TM helix of PBP1B is located between TM5 and TM1 of PgpB with extra contacts between TM1 of PgpB and the most deeply membrane-embedded region of the GTase domain of PBP1B. Additional contacts between the solvent-exposed periplasmic region of PgpB and the lowest part of the TPase domain stabilize this complex. This orientation places the 'donor strand'-binding region in the PBP1B GTase domain closest to the active site of PgpB (Fig. 8B). The ‘donor strand’ lipid II is the one whose oligosaccharide moiety is transferred to the C4 hydroxyl of the GlcNAc moiety in the ‘acceptor’ lipid II. Once a glycosyltransferase reaction is completed by transferring the donor strand (either lipid II or the growing glycan chain) onto the acceptor lipid II, the resulting C55-*PP* moiety has to be removed to allow the movement of the growing chain from the acceptor to the donor site. In the presented complex structure, the lipid carrier can easily access the active site of PgpB which would facilitate the release of C55-*PP* from the GTase domain permitting a faster polymerization of the peptidoglycan chain.

## Discussion

The major bi-functional PG synthases in *E. coli*, PBP1A and PBP1B, interact with monofunctional TPases (Bertsche et al., 2006, Banzhaf et al., 2012) and are activated by outer membrane-anchored lipoproteins (Typas et al., 2010, Paradis-Bleau et al., 2010), and the activation of PBP1B is modulated by components of the Tol system (Gray et al., 2015). PBP1B also interacts with other membrane embedded proteins including cell division protein FtsN (Müller et al., 2007) and FtsW (Leclercq et al., 2017), which has been shown to have lipid II flippase activity *in vitro* (Mohammadi et al., 2011). In this work we discovered a connection between the two C55-*PP* phosphatases PgpB and BacA with PBP1B, adding a new perspective on PG synthesis and pointing to a functional connection between the polymerization of PG and the recycling of the carrier lipid. Both phosphatases stimulated PBP1B in proteoliposomes, suggesting that the coupling of successive membrane steps in PG synthesis – lipid II polymerization and carrier lipid dephosphorylation – could be important in the cell. Indeed, the higher sensitivity of the *pgpB* mutant to cefsulodin suggests an impaired cellular functionality of PBP1B in the absence of PgpB.

We showed that the stimulatory effect of PgpB on PBP1B is likely due to a physical interaction between both proteins. We obtained contradictory results on a possible interaction of BacA with PBP1B, and BacA stimulated PBP1B to lesser extent than PgpB. These results are consistent with the idea that phosphatases generally stimulate PG GTases by converting their inhibitory C55-*PP* product into the less inhibitory C55-*P*, and that PgpB has an additional stimulatory effect associated with its specific interaction with PBP1B.

### How does PgpB stimulate PBP1B?

We observed the stimulation of PBP1B GTase by PgpB under two different conditions, at low PBP1B concentration in detergents (Fig. 5) and in the membrane environment of proteoliposomes (Fig. 6). In detergents, PBP1B shows higher activity under conditions where it dimerizes (Bertsche et al., 2005) and therefore it is possible that the interaction with PgpB stabilizes the more active dimer form of PBP1B. In a membrane context, PgpB stimulated PBP1B consumption of lipid II when incorporated in the same proteoliposomes (Fig. 6). This effect may be explained by the removal of C55-*PP* by the phosphatase, which might slow down PBP1B due to product inhibition as it happened in detergents (Fig. 7). Interestingly, the catalytically inactive version of PgpB also stimulated PBP1B in the membrane (Fig. 6A) while it failed to rescue inhibition by C55-*PP* in detergents (Fig. 7). Hence, the inactive PgpB(D211E) may accelerate the release of C55-*PP* from PBP1B (without hydrolysing it) only in the membrane system, allowing a faster polymerisation rate. The model structure of the PgpB-PBP1B complex obtained by *in silico* docking (Fig. 8) shows a close proximity between the active site of PgpB and the ‘donor’ lipid II binding site in PBP1B GTase domain, supporting such a coupled mechanism.

### Importance of the coupling between phosphatases and PBPs

C55-*P* is used to transport the precursors of several abundant cell envelope polymers across the cell membrane, for example those for the synthesis of PG and the O-antigen chains of LPS (Manat et al., 2014). *E. coli* maintains a pool of free carrier lipid of 1.5 × 10^5^ molecules per cell, 25% of these are in the 'active' C55-*P* form and 75% as C55-*PP* (Barreteau et al., 2009). The maintenance of the C55-*P* pool requires the efficient recycling of C55-*PP* by four phosphatases, BacA, PgpB, YbjG and LpxT (El Ghachi et al., 2005). The reason for this redundancy is unclear but BacA appears to have the highest activity (and importance) in the cell (El Ghachi et al., 2005) and LptX is a special case because it transfers the phosphate moiety form C55-*PP* to lipid A (Touzé et al., 2008b, El Ghachi et al., 2005).

Here we show that PgpB acts together with PBP1B and not PBP1A, forming a tight complex, which potentially could stimulate both enzymes. Complex formation with PBP1B could be a means to increase the efficiency of PgpB to dephosphorylate C55-*PP* immediately after the GTase reaction at the site where it is being released. We did not detect a substantial effect of PBP1B on the rate of C15-*PP* degradation by the phosphatases (Fig. 4). However, more experiments are needed to test the possibility that the degradation of C55-*PP* is faster when it is delivered directly from the polymerase than when it is freely diffusing in the membrane. The interaction between PgpB and PBP1B could improve the efficiency of PG polymerization in the cell membrane by preventing inhibition by locally high concentrations of C55-*PP* (Fig. 7). Our results are consistent with several possible mechanisms of stimulation, which are not exclusive. The association of both proteins may (i) accelerate the release of C55-*PP* from the active site of the GTase, (ii) prevent the inhibition of the GTase by the free pool of C55-*PP* on the periplasmic side of the cell membrane and/or (iii) cause an allosteric activation of PBP1B. While further experiments are needed to assess the contribution of each of these possibilities it is possible that C55-*PP* accumulates locally at sites of PG synthesis and, hence, its fast removal is achieved by the coupling of C55-*PP* phosphatases and PG synthases. Similar mechanisms might be used in other pathways involving the use of polyprenyl phosphates as lipid carriers for oligosaccharides across membranes.

PgpB has been described to have a dual role in the cell. The protein is not only involved in the dephosphorylation of C55-*PP*, but it also dephosphorylates phosphatidylglycerol-phosphate (PGP), the precursor of the most abundant anionic phospholipid in *E. coli*, phosphatidylglycerol (Tong et al., 2016). In fact, PgpB on its own is able to sustain phosphatidylglycerol synthesis in the absence of the other PGP phosphatases (Lu et al., 2011). This second activity raises the possibility that PgpB triggers the synthesis of anionic phospholipids at sites of PBP1B localization, and that the phospholipid composition could be another factor that regulates PG growth. Interestingly, it has been reported that peptidoglycan synthesis requires ongoing phospholipid synthesis (Rodionov & Ishiguro, 1996) and that this is likely due to disruption in lipid II transport when phospholipid synthesis is blocked (Ehlert & Höltje, 1996). In fact, it has been recently reported that lipid synthesis is a major determinant of bacterial cell size, independently of the stringent response by ppGpp (Vadia et al., 2017). Further studies are needed to decipher the possible role of phospholipids in the regulation of PG synthesis.

## Conclusions

To our knowledge we report here for the first time an interaction between a membrane-anchored oligosaccharide glycosyltransferase and a polyprenyl pyrophosphate phosphatase. The interaction between the PG synthase PBP1B and the membrane phosphatase PgpB stimulates PG synthesis in membrane systems, presumably due to the faster release of the carrier lipid C55-*PP* from the active site of the polymerase, and by preventing substrate inhibition by C55-*PP*.

## Conflict of interest

We have no conflict of interest.
