# Supplementary material for: Coupling of polymerase and carrier lipid phosphatase prevents product inhibition in peptidoglycan synthesis
Source: Cell Surf. 2018 Apr 19;2:1–13. doi: 10.1016/j.tcsw.2018.04.002 (PMC6053597; doi:10.1016/j.tcsw.2018.04.002)
Supplement: Supplementary data 1 [file mmc1.pdf]

Supplementary Information

Coupling of polymerase and carrier lipid phosphatase prevents product inhibition in peptidoglycan synthesis

Víctor M. Hernández-Rocamora[1*], Christian F. Otten[1*], Atanas Radkov[2^], Jean-Pierre Simorre[3], Eefjan Breukink[4], Michael VanNieuwenhze[2], Waldemar Vollmer[1#]

[1] Centre for Bacterial Cell Biology, Institute for Cell and Molecular Biosciences, Newcastle University, Richardson Road, Newcastle upon Tyne, NE2 4AX, UK.

[2] Department of Chemistry, Indiana University, 800 E. Kirkwood Avenue, Bloomington, Indiana 47405-7102, USA.

[3] Institut de Biologie Structurale, Université Grenoble Alpes, Grenoble, France.

[4]Membrane Biochemistry and Biophysics, Bijvoet Centre for Biomolecular Research, University of Utrecht, Padualaan 8, 3584 Utrecht, The Netherlands.

[^] Current address: Department of Biochemistry & Biophysics, University of California at San Francisco, UCSF Mission Bay Campus, 600 16th Street, San Francisco, CA 94143, USA.

[*] Contributed equally.

[#] Correspondence: w.vollmer@newcastle.ac.uk

Supplementary Information

Supplementary Table 1: Bacterial strains and plasmids.

Supplementary Table 2: Oligonucleotides.

Supplementary Figure 1: Weak interaction between BacA and PBP1B.

Supplementary Figure 2: Phosphatases do not affect the GTase rate in the presence of Triton X-100.

Supplementary Figure 3: C55-*PP* inhibits the GTase activity of PBP1B.

Supplementary References

Supplementary Table 1. Bacterial strains and plasmids.

Strain or plasmid	Relevant Genotype or Features	Source or Reference
**Strain**		
BL21(DE3)	F– *ompT hsdS*B(rB– mB–) *gal dcm* (DE3)	Novagen
C43(DE3)	F – *ompT hsdSB (rB- mB-) gal dcm* (DE3)	Millipore Sigma
BW25113	$lacI^q$ $rrnB_{T14}$ $\Delta lacZ_{WJ16}$ *hsdR514* $\Delta araBAD_{AH33}$ $\Delta rhaBAD_{LD78}$	(Datsenko & Wanner, 2000)
JW3029	BW25113 *ΔbacA::kan*	(Baba *et al.*, 2006)
JW1270	BW25113 *ΔpgpB::kan*	(Baba *et al.*, 2006)
JW5112	BW25113 *ΔybjG::kan*	(Baba *et al.*, 2006)
JW3359	BW25113 *ΔmrcA::kan*	(Baba *et al.*, 2006)
JW0145	BW25113 *ΔmrcB::kan*	(Baba *et al.*, 2006)
**Plasmid**		
pET28a-bacA	pET28a(+) derivative, for overproduction of N-His_6-BacA, encoding for an N-terminal thrombin cleavage site	This study
pET28a-pgpB	pET28a(+) derivative, for overproduction of C-His_6-PgpB	This study
pET28a-pgpB-mut	pET28a(+) derivative, for overproduction of C-His_6-PgpB D211E	This study
pET28a-ispU	pET28a(+) derivative, for overproduction of N-His_6-UppS, encoding for an N-terminal thrombin cleavage site	This study

Supplementary Table 2. Oligonucleotides.

**Name**	**Sequence 5' -> 3'**	**Description**	**Used for**
ispU_NdeI_for	gcgcgcCATATG*ttgtctgct actcaaccac*	[gcgcgc]-[NdeI]-[*ispU*; fwd]	pET28a His6-*ispU* cloning
ispU_XhoI_rev	gcgcgcCTCGAG*tcaggctg tttcatcaccg*	[gcgcgc]-[XhoI]-[stop *ispU*; rev]	pET28a His6-*ispU* cloning
bacA_NheI_for	gcgcgcGCTAGC*gatatgca ctcg*	[gcgcgc]-[NheI]-[bacA; fwd]	pET28a His6-*bacA* cloning
bacA_XhoI_rev	gcgcgcCTCGAG*ttaaaaga acacgaca*	[gcgcgc]-[XhoI]-[stop *bacA*; rev]	pET28a His6-*bacA* cloning
pgpB_NcoI_for	gcgcgcCCATGG*cacgttcg attgccagacgtac*	[gcgcgc]-[NcoI]-[pgpB; fwd]	pET28a His6-*pgpB* cloning
pgpB_XhoI_rev	gcgcgcCTCGAG*actttcttgt tctcgttgcgc*	[gcgcgc]-[XhoI]-[His6-TGAGATCCGGCTGC-stop; rev]	pET28a His6-*pgpB* cloning
pgpB_D211E_for	*gcattggccacgcga**g**ctggtagt agctacg*	gat → gag	pET28a His6-*pgpB* mutagenesis
pgpB_D211E_rev	*cgtagctactaccagct**c**gcgtgg ccaatgc*	gat → gag	pET28a His6-*pgpB* mutagenesis

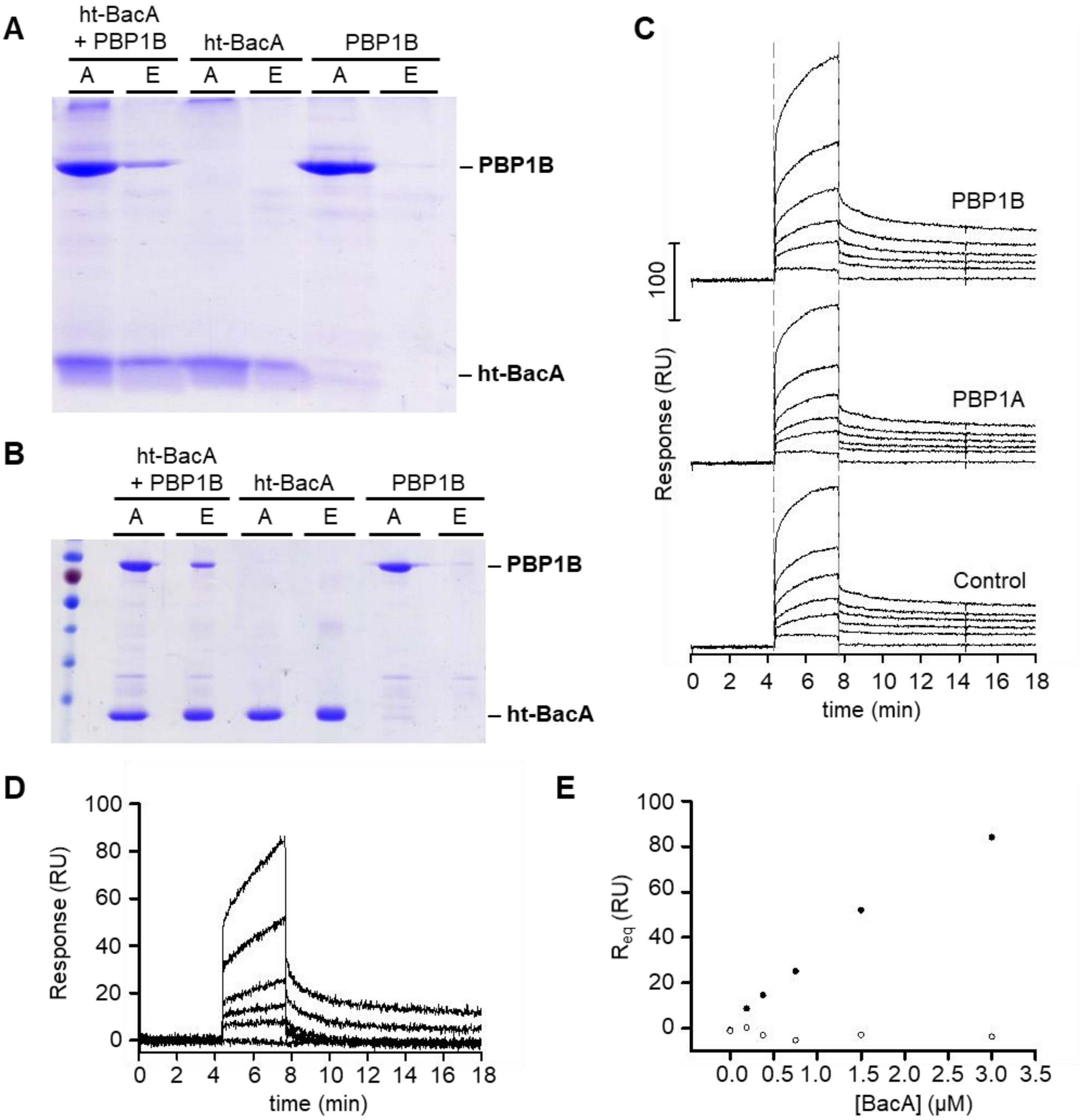

Supplementary Figure 1. Weak interaction between BacA and PBP1B. Oligohistidine tagged BacA (ht-BacA), PBP1B or both proteins together were incubated in the presence **(A)** or absence **(B)** of chemical cross-linker, followed by pull-down with Ni-NTA beads. ht-BacA pulled down PBP1B to Ni-NTA beads in both cases. Lanes: A, applied samples; E, elution samples. **(C)** PBP1B, PBP1A or no protein (control) was immobilised on a sensor chip with bound ampicillin. BacA was applied at concentrations of 3 µM, 1.5 µM, 0.75 µM, 0.375 µM or 0.163 µM. BacA bound slightly more to immobilised PBP1B than the control or PBP1A surfaces. **(D)** Sensorgrams of BacA applied to PBP1B surface with signal from the control surface subtracted. **(E)** Analysis of the titration data in panel C. The response at 5 min after injection minus the signal from the control surface was plotted against the concentration of BacA. White circles, PBP1A surface; black circles, PBP1B surface.

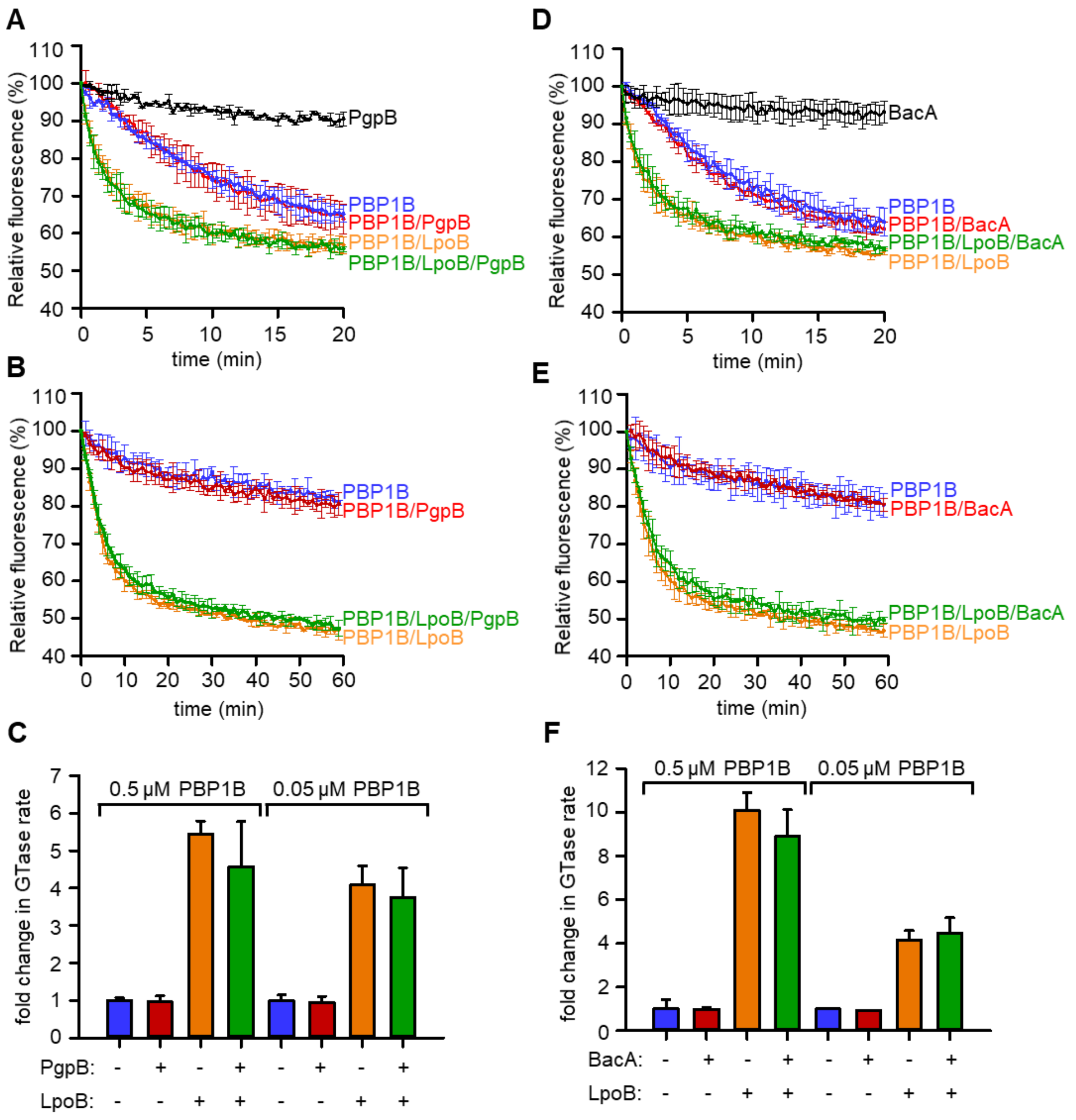

Supplementary Figure 2. Phosphatases do not affect the GTase rate in the presence of **Triton X-100.** The relative GTase rate of PBP1B at different concentrations was determined with dansylated lipid II as substrate. (**A**) 0.5 µM PBP1B in the presence or absence of PgpB and other proteins indicated. A PgpB alone control sample is included. (**B**) 0.05 µM in the presence or absence of PgpB and other proteins indicated. (**C**) Summary of the data shown in panels (**A**)and (**B**). (**D**) 0.5 µM PBP1B in the presence or absence of BacA and other proteins indicated. A PgpB alone control sample is included. (**E**) 0.05 µM in the presence or absence of BacA and other proteins indicated. (**F**) Summary of the data shown in panels (**D**) and (**E**). Each point in panels (**A**), (**B**), (**D**) and (**E**) represents the average and variation of 2 measurements. The values in panels (**C**) and (**F**) are mean ± standard deviation.

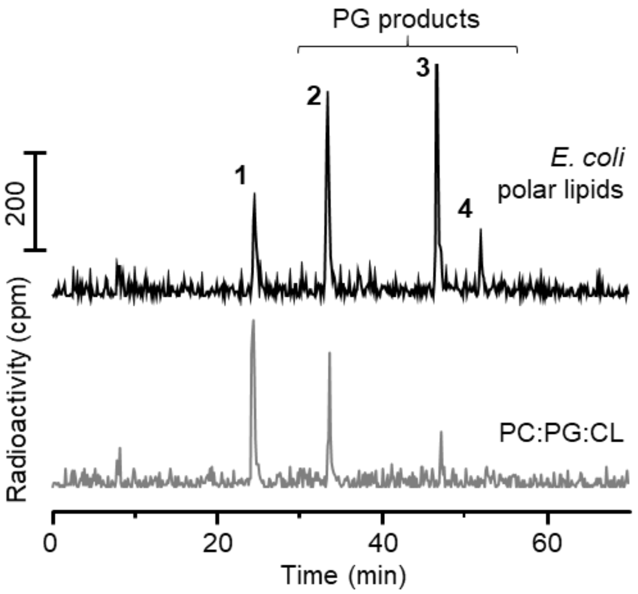

Supplementary Figure 3. PBP1B has lower activity in liposomes with artificial lipids. Representative chromatograms showing the activity of PBP1B reconstituted in proteoliposomes containing either natural *E. coli* polar lipid extract or a mixture of artificial lipids (PC:PG:CL). Radiolabelled lipid II was used as substrate and PG products were analysed by HPLC (as in Fig. 5A). The peak numbers relate to the muropeptide structures in Fig. 5D. The artificial lipid mixture contained DOPC:POPG:TOCL 70:20:10 (molar ratio). Both samples were assayed in 20 mM Tris/HCl pH 7.5 with 1 mM $MgCl_2$.

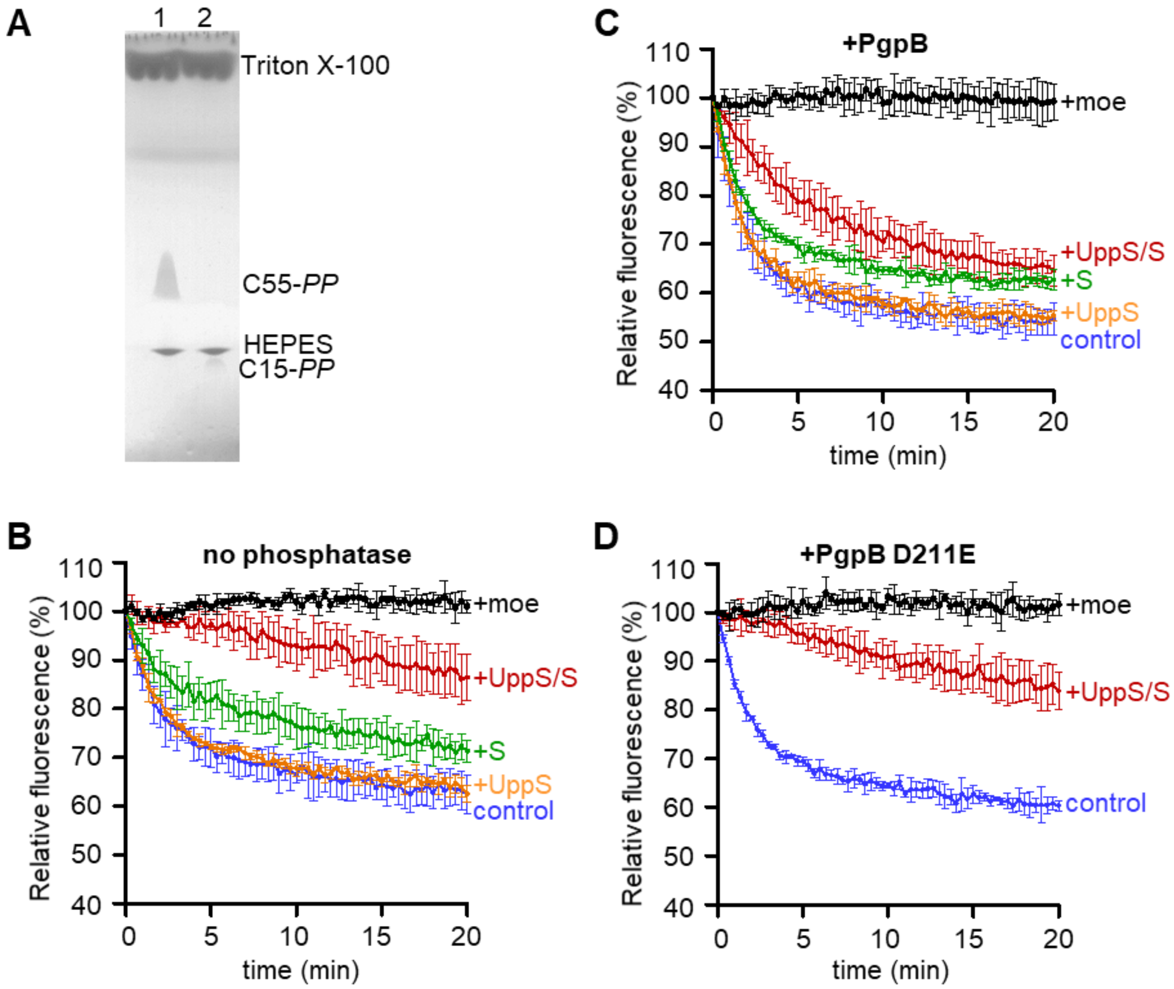

Supplementary Figure 4. C55-*PP* inhibits PBP1B glycosyltransferase (GTase) activity. **(A)** TLC analysis of the products of the UppS reaction with C15-*PP* and C5-*PP* (lane 1). Lane 2 shows a control with the substrates but no UppS. For C55-*PP* synthesis, UppS was added at 10 µM along with 2 nmol C15-*PP* and 20 nmol C5-*PP* per reaction and incubated at 25°C for 5 h. **(B)** Continuous fluorescence assay for PBP1B GTase activity in the presence (+Upps/S) or absence (control) of reactions with UppS and its substrates such as the one shown in **(A)**. Controls were performed using mock reactions including only UppS (+UppS) or only the substrates (+S). Moenomycin was added as a control to block PBP1B GTase activity (+moe). **(C)** shows the same assays as in **(B)** but performed in the presence of PgpB. **(D)** shows the same assays as in **(B)** but performed in the presence of PgpB inactive mutant D211E. The final concentrations of enzymes and substrates in **(B-D)** were: 0.5 µM PBP1B, 1 µM LpoB, 1 µM PgpB or PgpB D211E, 3.09 µM UppS, 33.3 µM C15-*PP*, 333.3 µM C5-*PP* and 10 µM dansyl-lipid II. GTase reactions were performed at 25°C. Each point represents average and variation of 3 measurements.

Supplementary References

Baba, T., Ara, T., Hasegawa, M., Takai, Y., Okumura, Y., Baba, M., Datsenko, K.A., Tomita, M., Wanner, B.L., and Mori, H. (2006) Construction of *Escherichia coli* K-12 in-frame, single-gene knockout mutants: the Keio collection. *Mol Syst Biol* **2:** 2006.0008

Datsenko, K.A. and Wanner, B.L. (2000) One-step inactivation of chromosomal genes in *Escherichia coli* K-12 using PCR products. *Proc Natl Acad Sci U S A* **97**: 6440-6445
